# Role of Penile Girth and Length in the Erect State in Reassuring Patients with Small Penis Anxiety

**DOI:** 10.5152/tud.2025.24133

**Published:** 2025-03-06

**Authors:** Sameh Fayek GamalEl Din, Nashaat Ismail Nabil, Mohamed A. Khalil, Amgad Elseginy, Mohamed Ahmed AbdElSalam, Ayman Mahmoud Ahmed Kamal, Ahmed Ragab

**Affiliations:** 1 Department of Andrology, Sexology & STDs, Cairo University Faculty of Medicine, Cairo, Egypt; 2 Department of Andrology, Sexology & STDs, Beni-Suef University Faculty of Medicine, Beni Suef, Egypt; 3 Hospital of Addiction and Pyschiatry, Cairo University Faculty of Medicine, Cairo, Egypt; 4 Ministry of Health & Population, Giza, Egypt

**Keywords:** Prostaglandin-E1, penile girth and penile length in the erect state, small penis anxiety

## Abstract

**Objective:**

The present study examined the impact of measuring penile girth and length in the erect state on reassuring individuals with small penis anxiety (SPA) in the flaccid state.

**Methods:**

This study included 200 potent men aged 25-40 years old. All candidates were assessed by the validated Arabic version of the International Index of Erectile Function (ArIIEF-5), Hospital Anxiety and Depression Scale (HADS), Beliefs about Penile Size, and Cosmetic Procedure Screening questionnaires. Participants were divided into 100 healthy participants and 100 participants with SPA. We measured penile length and girth in the flaccid state in participants with SPA. After 2 weeks, we measured penile length and girth in the erect state by injecting 0.25 cc diluted prostaglandin-E1 (PGE-1) in the corporeal bodies. Also, participants with SPA were reassessed by the ArIIEF-5 and the HADS in the erect state.

**Results:**

Penile length in the flaccid state was in the normal range 9.33 ± 0.81 cm that increased to 10.37 ± 0.89 cm in the erect state. The penile girth in the flaccid state was also in the normal range 8.08 ± 0.85 cm that increased to 9.33 ± 0.85 cm in the erect state. Significant decreases in the scores of HADS after PGE-1 injection were noted, denoting improvement in the patient’s quality of life. The ArIIEF-5 score insignificantly increased after re-counseling in the erect state. Despite a significant reduction in anxiety and depression levels after re-counseling in the erect state, yet, they did not show any correlation with the penile dimensions in the flaccid and the erect states.

**Conclusion:**

Although the current study failed to demonstrate significant correlations between penile dimensions in the erect state and the ArIIEF-5 and the HADS scores. Yet, there was significant decrease in HADS score and insignificant increase in ArIIEF-5 score denoting that men with SPA should be counseled in the erect state.

**Main Points**

- Body dysmorphic disorder should be differentiated from small penis anxiety (SPA).
- Although there were numerical improvements in the length and girth in the erect state, they did not show any correlation with improvement in erectile function.
- Any patient diagnosed with SPA should be counseled in the erect state, as our study demonstrated a significant reduction in the anxiety and depression scores after being properly counseled in the erect state.

## Introduction

Small penis anxiety (SPA) was defined as individuals with a normal-sized penis but who are overly anxious ab out its size with exclusion of micro-penis.^1^ A micro-penis has been identified as a penis <7.5 cm in erect length or <4 cm in flaccid length.^2^ Some men with SPA might be diagnosed with body dysmorphophobic disorder (BDD) focused on their genitals.^3^ Men who are anxious about penis size may link this anxiety to penile length, girth, or both. In addition, concerns may be present for both erect and flaccid penis sizes.^4^ Men with BDD are preoccupied with a perceived defect in their physical appearance that is not noticed or only minimally observed by others, causing significant distress or disorders in social, occupational or other important areas of functioning.^3^ diagnostic and statistical manual 5th edition (DSM-5) (American Psychiatric Association, 2013) added another criterion to DSM-IV, requiring that at some point during the course of BDD, the individual had performed repetitive behaviors or mental acts in response to preoccupied mind.^5^ Occasionally, BDD in men focuses on their genitalia, especially penis size.^6^^-^^8^ A man’s perception of his own penile size can impact self-confidence and a positive body image. Men who are self-conscious or suffer from severe preoccupation with penis size may experience anxiety-induced erectile dysfunction (ED) or other psychiatric disorders such as obsessive-compulsive disorder.^8^ The Arabic literature emphasizes that a longer penis is preferred; this idea is still embedded in many Arabic minds.^9^ Also, these men often look at the penis as a benchmark of masculinity.^9^ Studies had been conducted to evaluate penile dimensions among the average population and reported a normal adult penile flaccid length range of 8-10 cm and a stretched penile length range of 12-13 cm.^2^,^10^,^11^

In the same context, none of 67 patients complaining of a short penis were found to have a severely short penis.^12^ Similarly, a study conducted by Shamloul found that his patients had neither ED nor a short penis according to accurate measurements.^13^ The current study examined the impact of measuring penile girth and length in the erect state on reassuring individuals with SPA in the flaccid state.

## Material and Methods

The present study included 200 participants who attended the andrology clinic of Beni-Suef University Hospital and were worried about their penis size in the flaccid state from June 2022 to December 2023. The Review Board of Beni Suef University on 4th of October, 2020 approved the study protocol that conformed to the Helsinki Declaration 2013 ((FMBUREC/04102020)).^14^ All study participants provided informed consent after being properly counseled about the nature of the study with the necessity of evaluating participants with SPA in the erect state, which required injecting them with 0.25 cc diluted prostaglandin-E 1 (PGE1, prostin VR Pfizer). About 1 cc of prostin VR is diluted by 24 cc saline then 0.25 cc of the diluted prostin VR is withdrawn to be used for intracorporeal injection (ICI). Thus, 0.25 cc of diluted PGE1 contains 20 µg PGE1 to avoid the risk of priapism.

### Inclusion Criteria of the Participants

Potent men aged 25-40 years old who had SPA.

### Exclusion Criteria of the Participants

Any patient with BDD was excluded. Any patient with a history of endocrinological disease or congenital penile abnormalities that may affect penile dimensions, such as hypospadias, epispadias was excluded. Also, cases of Peyronie’s disease, past history of prostatic surgery, or cases who received testosterone replacement therapy for hypogonadism and ED were excluded.

### Inclusion Criteria of the Controls

They were age-matched, healthy, potent individuals recruited from the outpatient clinic, coming for fertility assurance and premarital check-up. They were recruited in alignment with the nomograms of a systematic review meta-analysis conducted by Veale et al.^15^

All participants were subjected to the following:

During the first visit, full medical and surgical histories were obtained. General and local examinations were performed. All participants were evaluated by the Arabic version of the International Index of Erectile Function (ArIIEF-5) to exclude ED (severe (5-7), moderate (8-11), mild to moderate (12-16), mild (17-21), and no ED (22-25).^16^ Also, they were initially evaluated by the Beliefs About Penile Size (BAPS) questionnaire to discriminate between participants who had concerns or were dissatisfied with their penile size and patients who were suffering from BDD (>20 in total shows body dysmorphia).^17^ Moreover, they were also initially evaluated by the Cosmetic Procedure Screening (COPS) questionnaire, as this test has a high sensitivity for diagnosing BDD (>40 in total shows body dysmorphia).^18^ Furthermore, all participants were evaluated by the Hospital Anxiety and Depression Scale (HADS) in the flaccid and the erect states to determine the impact of reassuring them about their erect penis size on their levels of depression and anxiety (0-7: normal, 8-10: borderline abnormal, 11-21: abnormal).^19^ Furthermore, the participants were evaluated again by the ArIIEF-5 in the erect state. Penile girth was estimated at the mid-penile shaft.^13^ Measurements of the penile girth in the flaccid state with the penis fully stretched after 3 successive stretches were performed.^20^ The penile length was defined as the linear distance between the symphysis pubis and the tip of the glans in the flaccid state.^13^ It should be noted that penile length in controls was also evaluated in the stretched state as we could not evaluate them in the erect state.^15^

The penile length was measured by holding the glans penis with the thumb and the index fingers and measurement was taken from the pubic ramus to the distal tip of the glans penis over the dorsal side. Firm pressure was applied on the pubic bone in a recumbent position.^13^ The measurements were executed by the same doctor immediately after penis exposure to minimize the effects of temperature.^13^ A structured interview was conducted where the following questionnaires were given to be answered: BAPS, COPS, HADS, and ArIIEF-5 for all participants. After 2 weeks, participants with SPA were asked to return for a second evaluation after injecting their penis with 0.25 cc prostaglandin-E 1 (PGE1, prostin VR Pfizer) and were observed until an erection was noticed.^21^ The penile dimensions were then measured in the erect state. After reassuring the candidates that their erect penis size was within the normal range, they were re-evaluated using only the HADS and ArIIEF-5 questionnaires in a structured interview.

### Statistical Methods

Data were statistically described in terms of mean ± standard deviation (±SD), median and range, or frequencies (number of cases) and percentages when appropriate. Because the groups were large enough, comparison between the study and control groups was done using the Student’s *t*-test for independent samples. Within-group comparison before and after the intervention was done using the paired *t-*test. Correlation between various variables was done using Pearson moment correlation equation for linear relation of normally distributed variables and Spearman rank correlation equation for non-normal variables/non-linear monotonic relation. Two-sided *P* values less than .05 were considered statistically significant. IBM SPSS ((IBM SPSS Corp.; Armonk, NY, USA) release 22 for Microsoft Windows was used for all statistical analyses.

### Sample Size Determination

Sample size calculation was done using the comparison of BAPS scores between patients with SPA and matched control subjects. Searching the literature failed to find any previous results that could be used to build up the sample size. Therefore, we performed a pilot study to obtain usable results. According to our pilot study, the mean ± SD of BAPS in participants with the SPA group was 5.4 ± 2.51, while in controls it was 7 ± 1.6. Accordingly, we calculated that the minimum proper sample size was 100 participants in each group to be able to detect a real difference of one unit in BAPS score with 80% power at *α* = 0.05 level using Student’s *t*-test for independent samples. Sample size calculation was done using Stats Direct statistical software version 2.7.2 for MS Windows, StatsDirect Ltd., Cheshire, UK.

## Results

**Figure 1.**
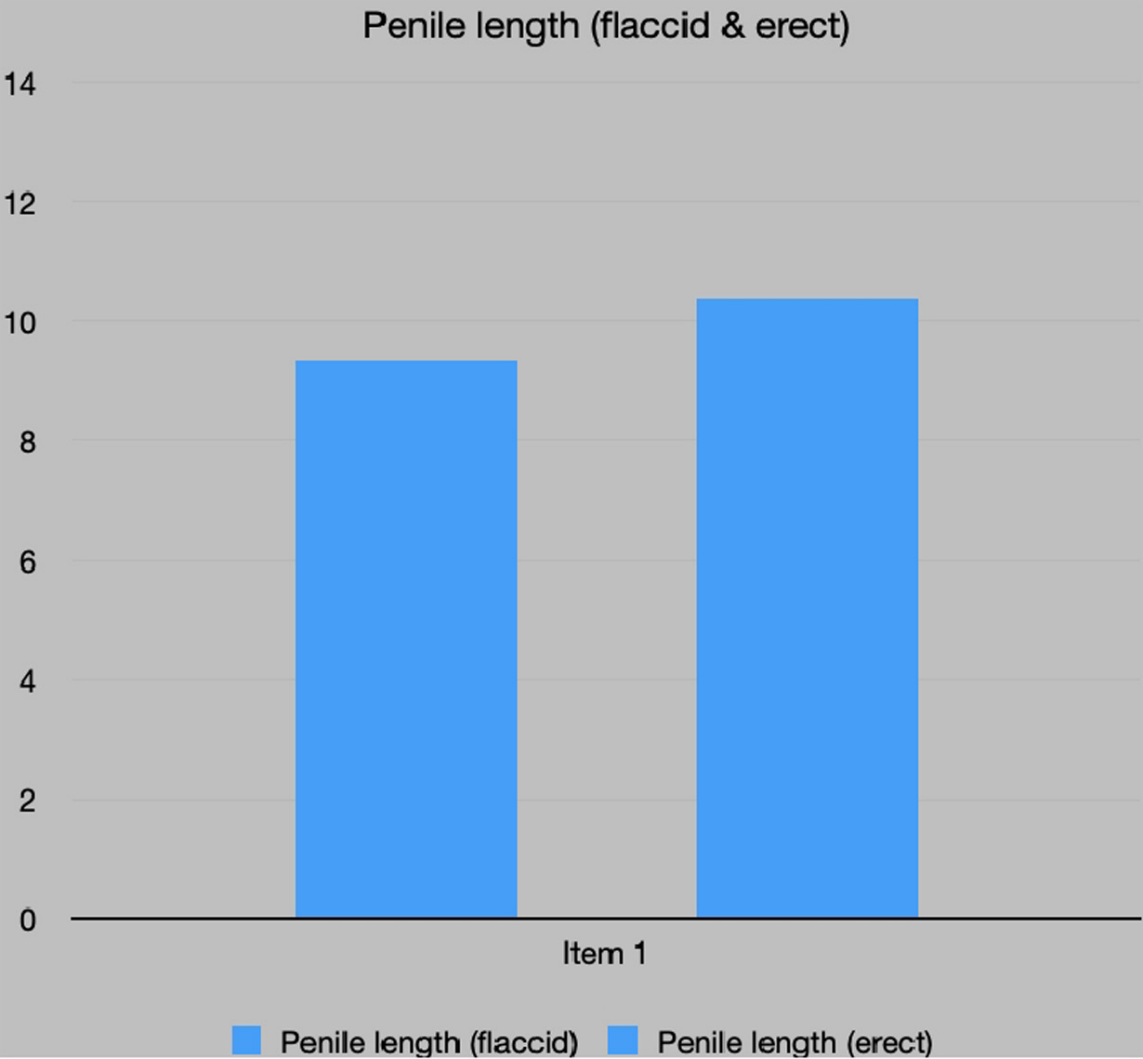
Histogram that shows changes in the penile length in the flaccid and erect states.

**Figure 2.**
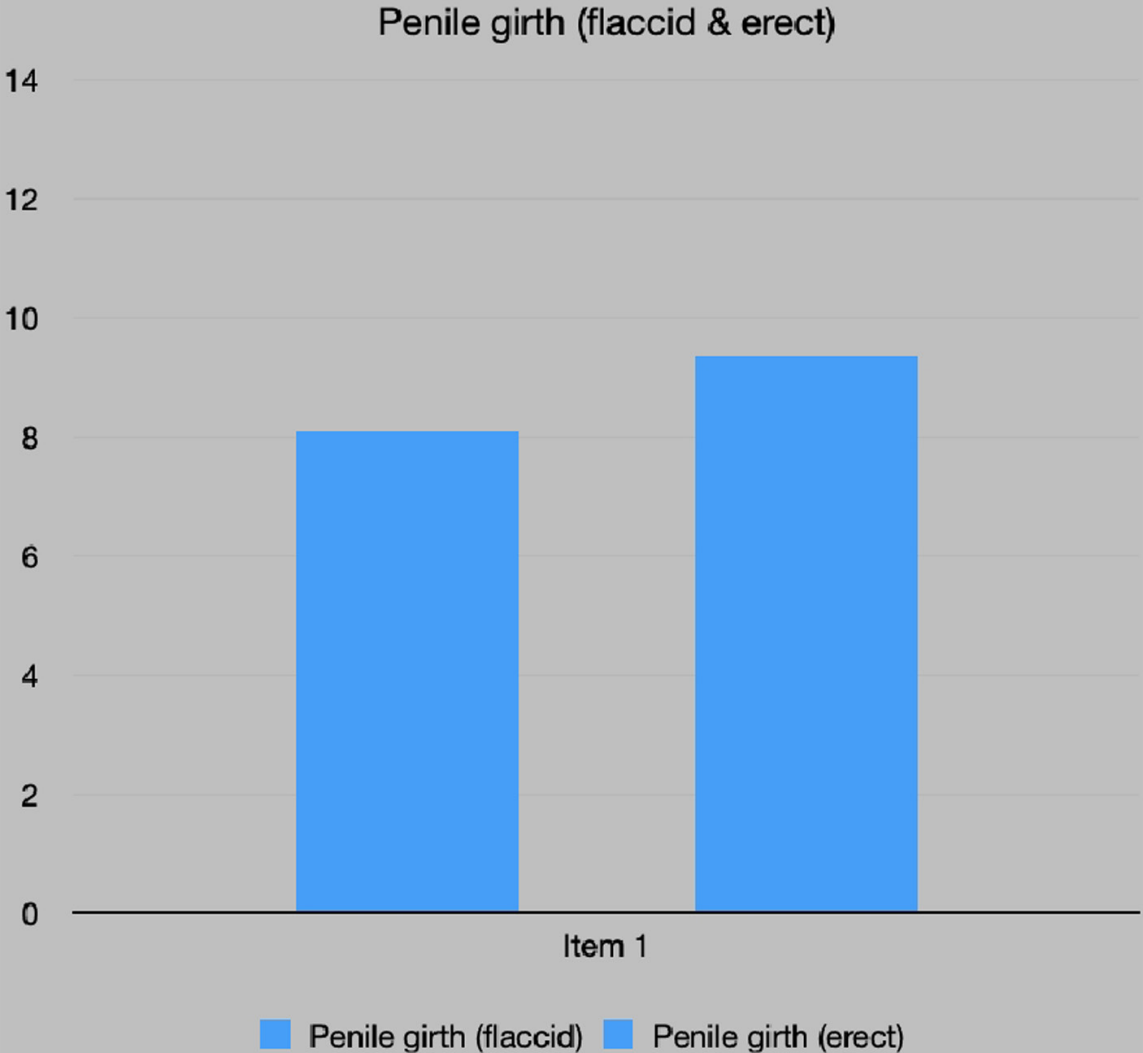
Histogram that shows changes in the penile girth in the flaccid and erect states.

**Figure 3.**
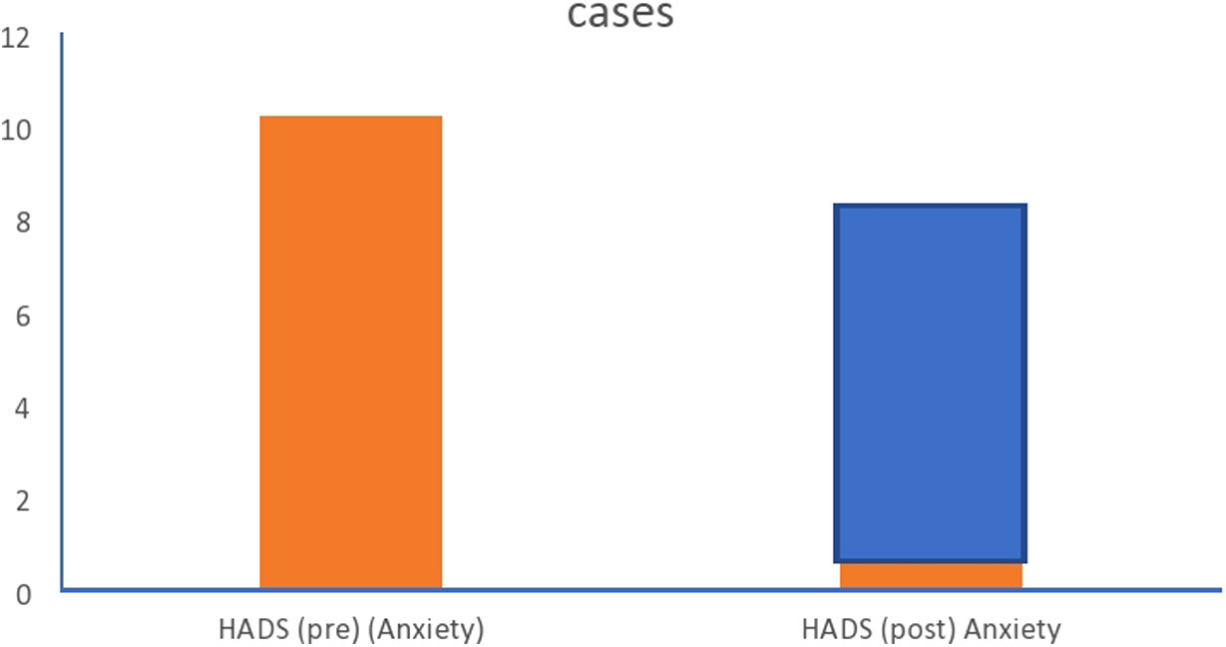
Histogram that shows improvement in the anxiety level in the patients in the flaccid and erect states.

The study recruited 200 participants. All participants were screened for BDD by COPS and BAPS. One hundred participants were healthy and served as controls. Four patients out of the 100 participants with SPA admitted to the clinic showed symptoms of BDD and were excluded. Socio-demographic characteristics of all participants were presented in Table 1. The age of all participants ranged from 25 to 40 years (Table 1). The penile length in the flaccid state was in the normal range 9.33 ± 0.81 cm (Table 2, Figure 1). In the erect state, the penile length increased after PGE-1 injection to become 10.37 ± 0.89 cm (Table 2, Figure 1). The penile girth in the flaccid state was also in the normal range 8.08 ± 0.85 cm (Table 2, Figure 2). The penile girth increased in the erect state to become 9.33 ± 0.85 cm (Table 2, Figure 2). Sixty-two participants (62%) with SPA were borderline anxious when counseled in the flaccid state (Table 3, Figure 3). Interestingly, 65 participants (65%) with SPA became normal after re-counseling in the erect state (Table 3, Figure 3).

**Figure 4.**
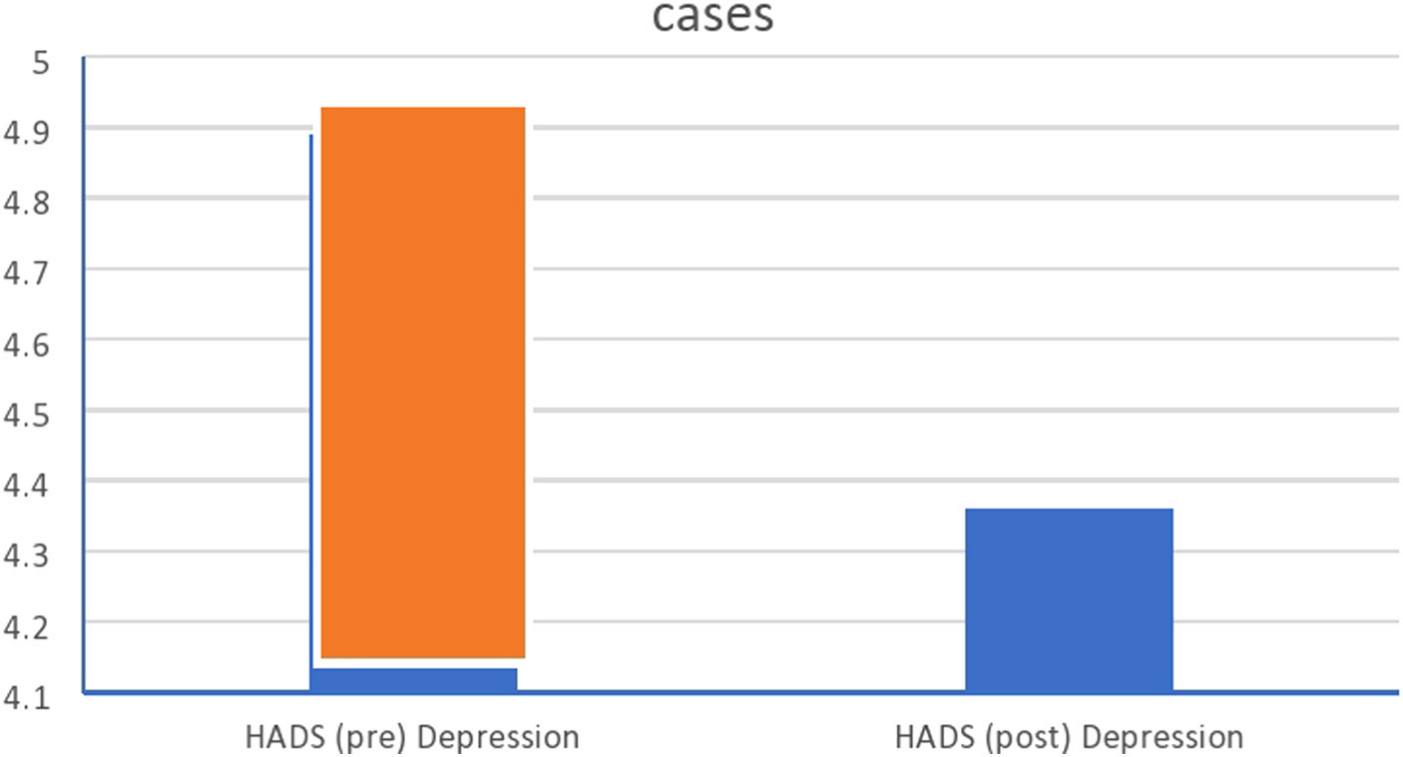
Histogram that shows improvement in the depression level in the patients in the flaccid and erect states.

Also, 14 participants with SPA and 11 participants with SPA were borderline abnormal and abnormal on the depression scale of the HADS, respectively (Table 3, Figure 4). Interestingly, 11 participants with SPA and 7 participants with SPA became borderline abnormal and abnormal on the depression scale of the HADS after re-counseling in the erect state, respectively (Table 3, Figure 4). Regarding scores of HADS pre and post PGE-1 injection, there were significant decreases in the scores denoting improvement of quality of life in participants with SPA (Table 4). Also, ArIIEF-5 score insignificantly increased after re-counseling in the erect state (Table 4). Despite a significant reduction in anxiety and depression levels after re-counseling in the erect state, yet, they did not show any correlation with penile dimensions in the flaccid and erect states (Table 5).

## Discussion

Our study showed that penile length and girth in the flaccid and erect states for the participants with SPA were in the normal range that could be seen in alignment with Ghanem et al and Habous et al.^20^^,^^22^ Our study showed significant decreases in the HADS scores after a 0.25 cc diluted PGE-1 injection, denoting improvement of quality of life in participants with SPA, coinciding with an insignificant increase in the ArIIEF-5 score. In fact, none of our participants with SPA had a short penis according to our measurements. Almost all participants with SPA in the current study overestimated the normal penis size. Most participants with SPA found their penile dimensions in the erect state quite helpful and reassuring in the current study, evidenced by significant decreases in HADS scores. Nevertheless, the current study failed to identify significant correlations between penile dimensions in the erect state and the ArIIEF-5 and the HADS scores. This may be attributed to the small sample size recruited in the current study.

Another underlying explanation for the negative correlation between penile dimensions in the erect state and the ArIIEF-5 score was the fact that participants with SPA already had good erections but they were worried about their size. This denotes the important role of the mental and psychic status of these particular groups of patients, necessitating proper counseling in the erect state, as evidenced by the significant reduction in HADS scores. Noteworthy, anxiety related to penis size could be frustrating and might affect self-esteem and relationships.^22^ The studied participants in the aforementioned study believed that their penises were unusually small, which was contradictory to the reality.^22^ Furthermore, Veale et al^23^ stated that men with BDD were more likely to have ED and less satisfaction with intercourse than men with SPA and controls. Consistently, Sharp et al stated that their studied men underwent augmentation surgery to achieve the ideal penis they were seeking, as they thought that their ideal penis, as well as what their penis “should be,” was significantly greater than their current girth and length.^25^ Also, the studied men in the previous study^24^ sought penile augmentation to increase their flaccid and erect length. However, a red flag should be considered in these patients, and they should be properly screened pre-procedure owing to the presence of some unrealistic expectations for their post-procedure length.^25^^-^^28^ To wrap up, the current study gains importance regarding the proper counseling for men with SPA in the erect state to avoid unrealistic expectations about their real penis size, thereby preventing unnecessary invasive penile augmentation procedures. Noteworthy, men who underwent penile augmentation might not be satisfied with the results due to increased anxiety associated with SPA, leading to complications.^29^ Admittedly, insufficient sample size of participants with SPA for proper statistical measurements during the required period could be identified as the main limitation of the current study.

Nevertheless, this study was a prospective study describing penile dimensions in the flaccid and the erect states in men seeking advice for SPA, which added strength and reliability to our results. Furthermore, we did not measure the length in the fully flaccid stretched state, as we thought that measuring the penile length in the erect state would be more reliable. This aligns with Habous et al^30^ who stated that erect penis measurements are more reliable for men being considered for treatment of SPA due to significant interobserver variability in estimating penile size in the flaccid state. Meanwhile, we measured the penis size in the controls in the fully flaccid stretched state as we could not expose them to invasive procedures such as ICI and the risk of developing priapism. Finally, the inability to use validated Arabic questionnaires for anxiety and depression evaluation, as well as BAPS and COPS, could be added as another limitation.

Although the current study failed to demonstrate significant correlations between penile dimensions in the erect state and the ArIIEF-5 and the HADS scores. Yet, there was significant decrease in HADS score and insignificant increase in ArIIEF-5 score denoting that men with SPA should be counseled in the erect state. Penile dimensions in the erect state can rectify any previous sexual over-expectations, relieve unnecessary anxiety concerning penis size, and decrease the desire to undertake augmentation procedures. Thus, accurate assessment of penile dimensions represents the cornerstone for evaluating men consulting for SPA and inhibiting their false belief that they have a small sized penis.

## Figures and Tables

**Table 1.** Comparison Between Demographic and Clinical Data Between Case and Control Groups

	Cases		Controls		*P*
	Mean	SD	Mean	SD	
Age	30.54	3.81	30.90	4.69	.604
Penile length (flaccid)	9.33	0.81	9.59	0.63	.102
Penile girth (flaccid)	8.08	0.85	13.19	0.73	.112
Penile length (erect)	10.37	0.89	–	–	–
Penile girth (erect)	9.33	0.85	10.43	0.56	.1022
COPS (pre)	16.23	7.45	22.70	8.83	1.155
COPS (post)	11.48	8.82	22.70	8.83	1.248
BAPS (pre)	7.07	4.86	10.55	4.00	.629
BAPS (post)	5.99	4.13	10.55	4.00	.575
HADS (pre) (anxiety)	10.18	1.61	3.41	2.45	.293
HADS (post) (anxiety)	7.38	2.56	3.41	2.45	.354
HADS (pre) depression	4.89	3.61	3.41	2.45	.436
HADS (post) depression	4.36	3.42	3.41	2.45	.421
IIEF-5 (pre)	22.84	2.29	23.10	1.05	.252
IIEF-5 (post)	30.54	3.81	30.90	4.69	.136

**Table 2.** Patients’ Ages and Their Penile Dimensions in the Flaccid and Erect States

	Mean	Standard Deviation	*P*
Age	30.54	± 3.81	–
Penile length (flaccid)	9.33	± 0.81	<.001
Penile length (erect)	10.37	± 0.89	
Penile girth (flaccid)	8.08	± 0.85	<.001
Penile girth (erect)	9.33	± 0.85	

**Table 3.** Frequency of Depression and Anxiety Before and After Counseling in the Flaccid and Erect States

Cases (n = 100)		Count	%
HADS (pre) anxiety	Normal	0	0.0
	Borderline abnormal	62	62.0
	Abnormal	38	38.0
HADS (post) anxiety	Normal	65	65.0
	Borderline abnormal	19	19.0
	Abnormal	16	16.0
HADS (pre) depression	Normal	76	76.0
	Borderline abnormal	14	14.0
	Abnormal	10	10.0
HADS (post) depression	Normal	82	82.0
	Borderline abnormal	11	11.0
	Abnormal	7	7.0

**Table 4.** Correlations Between Scores of HADS and ArIIEF-5 Before and After Injection of 0.25 cc PGE-1

Cases (n = 100)	Mean	Standard Deviation	*P*
HADS (pre) anxiety	10.18	1.61	<.001
HADS (post) anxiety	7.38	2.56	
HADS (pre) depression	4.89	3.61	<.001
HADS (post) depression	4.36	3.42	
ArIIEF-5 (pre)	22.84	2.29	.287
ArIIEF-5 (post)	23.08	0.87	

**Table 5.** Correlation Between Penile Dimensions in the Flaccid and the Erect States and Anxiety and Depression in the Participants

Cases (n = 100)		Penile Girth (Flaccid)	Penile Girth (Erect)
HADS (pre) (anxiety)	*r*	0.024	0.112
	*P*	.816	.268
HADS (post) (anxiety)	*r*	−0.047	0.069
	*P*	.644	.498
		3**Penile length (flaccid)**	3**Penile length (erect)**
HADS (pre) (anxiety)	*r*	0.114	0.094
	*P*	.260	.354
HADS (post) (anxiety)	*r*	0.069	0.074
	*P*	.494	.463

## Data Availability

>The data of this study is available upon request to the corresponding author.
